# Apple Pomace as a Source of Bioactive Polyphenol Compounds in Gluten-Free Breads

**DOI:** 10.3390/antiox10050807

**Published:** 2021-05-19

**Authors:** Dorota Gumul, Rafał Ziobro, Jarosław Korus, Marek Kruczek

**Affiliations:** Department of Carbohydrate Technology and Cereal Processing, Faculty of Food Technology, University of Agriculture in Krakow, Balicka 122 Street, 30-149 Krakow, Poland; rrziobro@cyf-kr.edu.pl (R.Z.); rrkorus@cyf-kr.edu.pl (J.K.); marekkruczek@gmail.com (M.K.)

**Keywords:** apple pomace, antioxidant activity, bioactive compounds, gluten-free bread, polyphenols

## Abstract

Gluten-free products based on starch and hydrocolloids are deficient in nutrients and do not contain pro-health substances. Therefore, they should be enriched in raw materials naturally rich in antioxidants, especially if they are intended for celiac patients, prone to high oxidative stress. Apart from the traditionally used pseudo-cereals, seeds, vegetables and fruits, innovative substrates such as the by-product (especially in Poland) dry apple pomace could be applied. The study material consisted of gluten-free bread enriched with apple pomace. The content of individual polyphenols, the content of total polyphenol and flavonoids, and also the antioxidant potential of the bread were determined by the UPLC-PDA-MS/MS methods. It was observed that apple pomace was a natural concentrate of bioactive substances from the group of polyphenols. In summary, gluten-free bread with 5% content of apple pomace showed the highest organoleptic scores and contained high levels of phenolic compounds. The values of total phenolic content, and the amounts of flavonoids, phenolic acids and phloridzin in this bread were 2.5, 8, 4 and 21 times higher in comparison to control.

## 1. Introduction

From a nutritional point of view, gluten is a mixture of storage proteins (monomeric gliadins and polymeric glutenins) present in mature wheat kernels [1], and this definition could be extended to homogenic proteins of rye (secalin), barley (hordein) and oats (avenin). Better diagnosis tools and the increasing self-awareness of consumers has resulted in a growing incidence of identified adverse reactions to these proteins in recent years. Among the disorders caused by gluten, celiac disease should be listed first. This is followed by ataxia and Dhuring disease, classified as autoimmune conditions, and wheat allergy, caused by overreaction of the immune system. Celiac disease (CD), which is the most frequent autoimmune enteropathy triggered by the ingestion of gluten in genetically susceptible individuals, is considered to be one of the most common human genetic disorders, occurring with a prevalence of about 1% of the total population worldwide [2,3]. It should be kept in mind that there is a strong relationship between gluten consumption and the occurrence of the above-mentioned conditions, so the only effective treatment of CD is a strict adherence to a gluten-free diet. A key role in gluten-free diet is played by gluten-free bread, which is usually inferior in nutritional value compared to traditional wheat and wheat-rye bread. The low nutritional quality of gluten-free bread (GFB) is caused by the lower content of protein, vitamins (folic acid, B vitamins) and minerals (Fe, Ca, Mg, Cu) [2,4]. Relevant studies indicate that up to 87% of adult patients are deficient in one or several vitamins (A, D, B6, B12) and minerals (Zn, Fe, Ca), and many celiacs have problems with the intake of calcium and vitamin D [5]. Moreover, GFB is also low in substances important from a physiological point of view (pro-health constituents), such as dietary fiber, which plays a substantial role in rational nutrition and the prevention of chronic diseases, such as hypertension, diabetes and cancer. Gluten-free products are deficient in nutritional and especially pro-health constituents, resulting in the occurrence of many disorders, such as osteoporosis, esophageal cancer, and infertility [5,6]. It should be noted that many authors [7,8,9,10] have indicated oxidative stress and cellular redox status as potential factors in the pathogenesis of celiac disease. People with CD usually exhibit significant oxidative stress and impaired performance of antioxidant enzymes (glutathione peroxidase, glutathione reductase, superoxide dismutase (SOD), and catalase), which form an important antioxidant barrier in the body, and are therefore prone to oxidant-antioxidant imbalance and DNA damage. The state of oxidative stress in celiac patients, measured by the level of oxidative DNA damage, could be minimized by the use of antioxidants (e.g., vitamin E and especially polyphenols) in the diet, which, among other things, would diminish the risk of cancer development. Therefore, it is important to create new recipes for gluten-free breads containing ingredients rich in natural antioxidants (polyphenols). Special attention should be given to gluten-free additives that are safe, contain large amounts of nutrients, especially pro-health constituents (polyphenols), and can be acquired in large quantities at a reasonable cost. It seems that dried apple pomace fulfills all of these requirements. Pomace is microbiologically stable in its dried form and consists of a heterogeneous mixture of various morphological elements of apples [11]. Therefore, it could be regarded as a concentrate of various pro-health constituents, mainly polyphenols, with anticancer, anti-inflammatory, antibacterial, and antiviral properties [12,13]. Apple pomace is rich in endogenous polyphenols, such as phenolic acids (especially chlorogenic acid), flavonoids (catechins, epicatechins) and dihydrochalcone (phloridzin) [14,15,16,17]. Its use in gluten-free bread production should have a significant influence on its antioxidant potential, and thus on its pro-health value, especially in celiac patients [18,19,20]. Thus, it can be suggested that gluten-free products enriched with antioxidants derived from apple pomace may become a potential product for overcoming oxidative stress, which is commonly found in people with celiac disease, especially as the numer of celiac patients is increasing each year.

Taking into account market aspects, economists expect that this sector could be one of the most profitable branches of the food industry [21]. This is why the study on the fortification of gluten-free products with natural supplements seems to be within the current trend of world research [18,19,20].

Therefore, the aim of the research was to analyze the total phenolic content and total flavonoid and antioxidant activity, as well as the quality and quantity of phenolic compounds in gluten-free breads with different contents of apple pomace (5; 10 and 15%). Additionally, the aim was to characterize apple pomace as a natural concentrate of bioactive substances belonging to the group of polyphenols.

## 2. Materials and Methods

### 2.1. Chemicals

Methanol, ascorbic acid, acetic acid, formic acid, and acetonitrile were purchased from Sigma-Aldrich (Germany). Quercetin, phloridzin, phloretin, catechin, epicatechin, procyanidin and phenolic acids were purchased from Extrasynthese (Genay, France). Folin-Ciocalteu’s phenol reagent, gallic acid, Trolox (Tx), rutin, and ABTS (2,2′-azino-bis(3-ethylobenzothiazoline-6-sulphonic acid)-diamonium salt) were purchased from Sigma-Aldrich (Hamburg, Germany).

### 2.2. Materials

The materials in this manuscript were gluten-free breads with different shares of apple pomace (5, 10, and 15%). The following abbreviations are used in the tables and figure—Control (control bread), GFB5AP (gluten-free bread with a share of 5% apple pomace), GFB10AP (gluten-free bread with a share of 10% apple pomace), GFB15AP (gluten-free bread with a share of 15% apple pomace). The apple pomace originated from apple concentrate made from apples of a multivarietal mixture of an autumn–winter seasonal group, from the production line at the Fruit and Vegetable Processing Plant HORTINO Leżajsk. The apple pomace, after oven-drying under industrial processing conditions, was twice milled in a laboratory grinder for 5 s at 7000 rpm (Grindomix, GM 200, Haan, Germany), and then in a laboratory hammer mill Lab Mill 3100 (16,800 rpm; Perten, Stockholm, Sweden).

### 2.3. Methods

#### 2.3.1. Bread Preparation

Control bread was baked according to the following recipe: corn starch 432 g, potato starch 108 g, freeze-dried yeast 27 g, guar gum 9 g, pectin 9 g, sucrose 10.8 g, salt 9 g, canola oil 16.2 g, water 558 g. In the other samples, part of the potato and corn starch (5, 10 and 15%) was replaced with an appropriate amount of apple pomace (5% apple pomace—27 g; 10%—54 g; 15%—81 g) All ingredients were mixed for 5 min (Laboratory Spiral Mixer SP 12, Diosna, Osnabrück, Germany). The dough was fermented for 15 min at 35 °C and relative moisture level 80%. After initial proofing, the dough was divided into 250 g pieces in greased baking pans and fermented for another 20 min under the above-mentioned conditions. The bread was baked at 230 °C for 30 min in an electric oven MIWE Condo type CO 2 0608 (MIWE GmbH, Arnstein, Germany). The loaves were removed from the pans, cooled, sliced, and air dried. Ground bread (crumbs with diameter below 1 mm) was stored in polyethylene bags for further study. Each formulation was baked in two independent batches, with five loaves in each batch.

#### 2.3.2. Antioxidant Content and Antioxidant Activity

The following analyses were performed on each sample of gluten-free bread that contained a share of apple pomace (additionally in apple pomace):

Antioxidant constituents and antioxidant activity were determined using ethanol extracts. An amount of 0.6 g of the sample was dissolved in 30 mL 80 g/100 g ethanol, shaken in darkness for 120 min (electric shaker: type WB22, Memmert, Schwabach, Germany), and centrifuged (15 min, 1050× *g*) in a centrifuge (type MPW-350, MPW MED. Instruments, Warsaw, Poland). The supernatant was decanted and stored at −20 °C for further analysis [22].

Determination of total polyphenol content (TPC) was performed by spectrophotometric method using Folin-Ciocalteu reagent (with F-C reagent), according to the method described by Singleton, Orthofer, and Lamuela-Raventós [23], and the content of flavonoids was evaluated using a spectrophotometrical method, according to the method described by El Hariri, Sallé, and Andary [24]. The results of TPC are expressed as mg gallic acid/100 g dry matter (d.m.) of sample. The results of flavonoid determination are expressed as mg rutin/100 g d.m. of sample.

Additionally, antioxidant activity was assessed using analytical methods with ABTS (2,2′-azino-bis(3-ethylobenzothiazoline-6-sulphonic acid)-diamonium salt) [25]. The results of antioxidant activity are expressed as TEAC (Trolox Equivalent Antioxidant Capacity—mg Trolox/g d.m. of sample).

#### 2.3.3. Determination of Individual Polyphenols by UPLC-PDA-MS/MS

1. Extraction

Samples (1 g) were extracted using 10 mL of reagent (3 mL methanol of a purity level of HPLC, 7 mL distilled water, 0.2 g ascorbic acid, 0.1 mL acetic acid). Extraction was carried out twice by incubating for 20 min under sonication (Sonic 6D, Polsonic, Warsaw, Poland) and mixing every 5 min. The suspension was then centrifuged at 19,000× *g* for 10 min and the supernatant was filtered through a 0.20 μm Hydrophilic PTFE membrane (marble filter Sampility Millex, Merck, Darmstadt, Germany) and used directly for analysis.

2. Assay

Phenolic compounds were measured using an Aquity Ultra Performance liquid chromatograph equipped with a Binary Solvent Manager (BSM), Sample Manager (SM) in combination with a PDA detector and a quadrupole time-of-flight (Q-TOF) detector (Waters, Manchester, UK). The extract samples (0.01 mL) were eluted according to a linear gradient. Analysis was performed on a 2.1 × 100 mm BEH C18 UPLC column containing particles of 1.7 μm (Waters, Manchester, UK). Isocratic elution was chosen as the gradient elution mode: 2 g formic acid/100 mL in water (A) and acetonitrile (B) as mobile phase at 0.45 mL/min. The mobile phase consisted of solvent A (2% formic acid) and solvent B (100% acetonitrile). The program began with isocratic elution with 99% solvent A (0–1 min), and then a linear gradient was used until 12 min, reducing solvent A to 0%; from 12.5 to 13.5 min, the gradient was returned to the initial composition (99% A), and then it was held constant to re-equilibrate the column. The column temperature was 30 °C, and the injection volume was 5 μL. The operating parameters of the mass detector were as follows: 2.5 kV capillary voltage and 30 V cone sample voltage. The ion source and desolvation temperatures were 130 °C and 350 °C, respectively. Nitrogen with a flow rate of 300 L/h was used as the carrier gas. Analyses were performed in full scan mode over the range 100–1500 *m*/*z*, with a tolerance of 0.001 Da and a resolution of 5000. The internal reference standard, leucine, was continuously pumped through a lockspray reference channel. Chromatograms were analyzed using a baseline peak (BPI) calibrated to 12,400 cps (100%). Data were collected and analyzed using MassLynx v. 4.1 software (Waters). Anthocyanins were analyzed in positive ion mode, and other polyphenols were analyzed in negative ion mode. Their identification was carried out by comparing maximum UV absorption spectra, molecular weight defined as mass-to-charge ratio, retention times, and fragmentation spectra with the available literature data. The degradation spectra were obtained by collision-induced dissociation (CID) in tandem mode. The collision energy was selected individually for each of the analyzed substances. Characteristic UV spectra were collected at the following wavelengths: λ = 320 nm—phenolic acids; λ = 360 nm—flavonols; λ = 280 nm—flavan-3-ols; λ = 340 nm—flavones. Retention times and spectra were compared with those obtained for pure standards. Quantification of phenolic compounds was carried out using external calibration curves, using standard compounds selected on the basis of the target analyte/structure standard (chemical structure or functional group). The calibration curve for p-coumaric acid was used for the quantification of 3-p-coumaroylquinic acid. performed in the concentration range of 0.05 to 5 mg/mL. The correlation coefficient was R^2^ ≤ 0.9998. Chlorogenic, cryptochlorogenic, and neochlorogenic acids were quantified according to an in-house standard was used for the quantification performed in the concentration range of 0.05 to 5 mg/mL. The correlation coefficient was R^2^ ≤ 0.9998. (+) catechin, (−) epicatechin, and procyanidin B2 were quantified according to an in-house standard, performed in the concentration range of 0.05 to 5 mg/mL. The correlation coefficient was R^2^ ≤ 0.9998. Calibration curves for 3-*O*-rutinoside, 3-*O*-glucoside and 3-*O*-galactoside of quercetin were used for quantification of quercetin derivatives, performed in the concentration range of 0.05 to 5 mg/mL. The correlation coefficient was R^2^ ≤ 0.9998. For the quantification of isorhamnetin derivatives, 3-*O*-rutinoside and 3-*O*-glucoside of isorhamnetin were used, respectively, performed in the concentration range of 0.05 to 5 mg/mL. The correlation coefficient was R^2^ ≤ 0.9998. All determinations were performed in duplicate (*n* = 2). The results are expressed as mg/100 g d.m. [26].

#### 2.3.4. Organoleptic Analysis

The breads were evaluated in accordance with a Polish standard [27] by a 15-person panel with proven sensory sensitivity. There were eight woman and seven men in this group, aged 21–48 years. The analyses were carried out in a laboratory designed and equipped in accordance with PN-ISO 8589 (1998) (PN-ISO 8589, 1998) [27]. The following traits were evaluated: external appearance (maximum 5 points), color (maximum 3 points), thickness (maximum 4 points) and other crust characteristics (maximum 4 points), elasticity (maximum 4 points), porosity (maximum 3 points) and other crumb characteristics (maximum 3 points), and smell and taste (maximum 6 points).

#### 2.3.5. Statistical Analysis

The experimental data were subjected to analysis of variance (Duncan’s test), at the confidence level of 0.05, by the use of software Statistica v. 8.0 (Statsoft, Inc., Tulsa, OK, USA). All measurements were performed at least in duplicate. Correlation coefficient was measured with the use of Statistica 8.0 PL.

## 3. Results and Discussion

### 3.1. Apple Pomace Characteristics

Table 1 shows the total phenolic content (TPC), total flavonoids and antioxidant activity of apple pomace. It can be observed that total polyphenol content reaches 89.4 mg gallic acid/100 g d.m. (Table 1). In the study of Candrawinata et al., [28] concerning apple pomace, the total phenolic content was 118.6 mg gallic acid/100 g d.m, while Bai et al., [29] reported a value of 62.7 mg gallic acid/100 g d.m. According to Adil et al. [30], total polyphenol content expressed in the same units was 47 mg gallic acid/100 g d.m, while Leyva-Corral et al., [17] noticed much higher levels, at 324.2 mg gallic acid /100 g d.m. Persic et al., [31] determined the total polyphenol content in apple pomace to be in the range 19–50 mg gallic acid/100 g d.m. In this context, it can be stated that the total polyphenol content in the analyzed apple pomace was within the range established by other authors, and the slight changes could be due to its origin. Ćetković et al., [14] reported that the broad range of determined polyphenols was largely a result of different apple varieties. At the same time, they observed that the amount of polyphenols could change by up to 30% within a given variety, depending on the year of harvesting. Such changes could consequently influence the level of polyphenols in apple pomace. Moreover, the values of TPC in plant materials are known to be dependent not only on the extraction conditions (type of medium, temperature, pH, time), but also on the way in which the results are expressed (e.g., different type of phenolic compound used to calculate the level of polyphenols) [28,29,30]. According to Rabetafika et al., [15], extraction using 60 or 70% acetone results in a better yield of polyphenols compared to extraction using 50% methanol. In the study of Krasnova and Seglina [32], the seasonal group of apples was also an important factor. It was observed that apple pomace derived from apples harvested in late winter contained almost twice as much TPC as that from apples produced in the autumn and winter seasons.

The total content of flavonoids in apple pomace was in the range 94.3 mg rutin/100 g d.m. (Table 1). According to Ćetković et al., [14], the content of flavonoids in apple pomace could vary between 45–119 mg rutin/100 g d.m. Krasnova and Seglina [32] also determined flavonoid content to be in the range of 240 to 685 mg catechin/100 g d.m., but they used catechin to express these compounds, and therefore we cannot compare our study to theirs.

Apart from determining total polyphenol content and flavonoids on the basis of spectrophotometric methods, UPLC-PDA-MS/MS analysis of the profile of individual phenolic compounds present in the apple pomace was performed. This made it possible to observe the presence of four groups of phenolic compounds in the analyzed samples: flavonols, flavan-3-ols, dihydrochalcons and phenolic acids (Table 2).

The total content of phenolic compounds determined using the UPLC-PDA-MS/MS methods was 118.7 mg/100 g d.m. (Table 2). Leyva-Corral et al., [17] reported the level of identified polyphenols to be 114.54 mg/100 g d.m., and Ćetković et al. [14] established a content of 69.2–147.4 mg/100 g d.m.

Among the phenolic compounds present in apple pomace, flavonols are the most abundant group, especially the derivatives of quercetin (Table 2), among which quercetin-3-*O*-galactoside—22.55 mg/100 g d.m., quercetin 3-O-rhamnoside—19.21 mg/100 g d.m. and quercetin-3-*O*-xyloside—13.91 mg/100 g d.m. predominated. Earlier reports on apple pomace showed the level of quercetin-3-*O*-glucoside to range between 28.6–61 mg/100 g d.m [14] or 52.1–68.1 mg/100 g d.m. [15], which is significantly higher than the value of 5.88 mg/100 g d.m observed in this study (Table 2).

Phenolic acids form another group of phenolic compounds present in substantial amounts in apple pomace, represented mainly by chlorogenic acid (20.55 mg/100 g d.m.) (Table 2). Other important phenolic acids include cryptochlorogenic acid—1.03 mg/100 g d.m. and p-coumaroylquinic acid—0.16 mg/100 g d.m. (Table 2). The content of p-coumaroylquinic acid was also reported to be 0.18 mg/100 g d.m. by Kammerer et al. [16]. The level of chlorogenic acid has previously been reported to be 1.43 mg/100g d.m. [16]; 3–17, 6 mg/100 g [14], and 41.55 mg/100 g d.m. [17].

Other very important groups of phenolic compounds present in apple pomace include flavan-3-ols and dihydrochalcons. Among flavan-3-ols, special attention should be given to catechin—1.44 mg/100 g d.m., procyanidin B2—2.61 mg/100 g d.m., and epicatechin—0.76 mg/100 g d.m. (Table 2). Previous reports have determined the catechin content to be: 0.94–1.4 mg/100 g d.m. [15]; 0.24 mg/100 g d.m. [16] and 1.7–12.7 mg/100 g d.m. [14]. The level of epicatechin in apple pomace has previously been reported to be: 2.4–17.3 mg/100 g d.m., 0.93 mg/100 g d.m., 14–19 mg/100 g d.m., and 12.23 mg/100 g d.m. [14,15,16,17]. The content of procyanidin B2, as assessed by other authors, was: 0.93 mg/100 g d.m., and 9.3–16 mg/100 g d.m. [15,16]. Among the dihydrochalcons, phloridzin was prevalent, being present in the amounts 15.52 mg/100 g d.m (Table 2). According to Leyva-Corral et al. [17], its content was equal to 17.97 mg/100 g d.m., while Ćetković et al. [14] determined it to be in the range of 0.7–8.5 mg/100 g d.m.

Our results may differ from those of other authors on the basis of many factors, such as apple variety, climatic and soil conditions, agrotechnical conditions, technology of pomace production, and the method of sample preparation for the chromatographic analysis [31,33]. All these factors may be responsible for discrepancies between the obtained results and the results obtained by the cited authors. Rana et al. [34] stated that drying apple pomace does not significantly affect the phenolic content in industrial apple pomace, and the most economically beneficial drying method is oven drying, which was used in this work. In the study of Rana et al. [34] on dried apple pomace, the polyphenols were determined in the range of 100–331 mg gallic acid/100 g, and the content of flavonoids was 15–99 mg quercetin/100 g, except that a different extraction method was used for these compounds, hence the high amount of TPC in the apple pomace compared to our study. Nevertheless, the previously cited authors [14,15,16,17,28,29,30,31,32,33,34] noted that the primary phenolic compounds in apple pomace were chlorogenic acid, catechin, epicatechin, quercetin derivatives, procyanidin B2, and phloridzin, with the latter compound being unique. It is only present in dried apple pomace and is a specific marker for this type of pomace [33]. The results of the above-mentioned authors regarding the dominant phenolic compounds in the analyzed apple pomace were confirmed in this work. The large number of the polyphenols in apple pomace makes it possible to state that it is a rich source of pro-health compounds, with anticancerogenic, hypoglycemic, hypotensive, antibacterial, antiviral and anti-inflammatory effects [12,13]. Among these bioactive compounds, special attention should be given on the one hand to catechin, procyanidin B2, epicatechin, which are characterized by a strong antioxidant effect and inhibit LDL oxidation (in vitro studies), and on the other to chlorogenic acid, which dominates as an anticancerogenic component. Quercetin, as a strong antioxidant, has a potential preventive effect on the development of many types of cancer (especially hormone-dependent cancers) and heart diseases, and is a factor inhibiting the development of colorectal cancer cells and adenocarcinomas. Phloridzin, which could be regarded as a marker of apple pomace, has an antidiabetic effect and reduces postprandial glycemia [35,36,37,38].

Taking into account celiac patients, it is important to note the high antioxidant activity of apple pomace. The high polyphenol content in apple pomace results in a significant antioxidant activity in this type of pomace (TEAC 9.30 mg Tx/g d.m., 0.036 mmol Tx/g d.m.) (Table 1). Additionally, Gorjanović et al., [39] determined the high antioxidant activity of dried apple pomace to be in the range of 0.034 to 0.1 mmol Tx/g. However, it should be emphasized that the antioxidant potential of apple pomace is not only a result of the presence of polyphenols, but is also influenced by other compounds with antioxidant properties (vitamin C, E, beta-carotene) and minerals, the contents of which were not determined in this study. The results of other authors reveal that apple pomace contains the vitamins C, E and beta-carotene [33], as well as high levels of macro- and micro-elements (K—4.49 g/kg, Ca—1.50 g/kg, P—1.49 g/kg, Mg—0.45 g/kg, Fe—91.8 mg/kg, Mn—8.75 mg/kg, Zn—6.90mg/kg, Cu—1.36 mg/kg) [40]. The latter components (Cu, Zn and Mn), especially, have the ability to stimulate antioxidant enzymes in our organism, thus guaranteeing their proper function. Apple pomace is therefore a natural and very valuable concentrate of endogenous antioxidants that is able to provide valuable antioxidant properties to the fortified gluten-free products. We suggest that gluten-free products fortified with apple pomace could eliminate the overproduction of free radicals in the organisms of celiac patients.

In the following steps of this study, we analyzed bread baked with apple pomace to verify the hypothesis that this type of addition, which could be regarded as a natural concentrate of antioxidants from the polyphenol group, will provide them with pro-health value.

### 3.2. Profile of Phenolic Compounds in Gluten-Free Bread Enriched with Apple Pomace

Table 3 demonstrates the quantity of total polyphenols and flavonoids in gluten-free bread enriched with apple pomace, as well as the antioxidant activity of these products.

It can be observed that total polyphenol content (TPC) significantly increased in gluten-free bread after the addition of apple pomace (2.5–20 times) in comparison to control. The change was parallel to the level of applied fruit component, with the greatest increase being provided with the 15% addition (Table 3). TPC in control gluten-free bread (1.02 mg gallic acid/100 g d.m. or, equivalently, 2 mg catechin/100 g d.m.) is probably due to the occurrence of the Maillard reaction, because its products, according to previous reports [41] could react with Folin-Ciocalteu reagent [42]. Moreover, according to Katina et al. [43], the fermentation process could increase the content of total polyphenols in bread. The results are in good agreement with previous data concerning the level of total polyphenols in GFB (control) [44], which reported a value of 5.2 mg catechin/100 g d.m. The above-mentioned research on gluten-free bread with the addition of blackcurrant and strawberry seeds showed increases in TPC in the range between 92% and 1265% compared to control [44].

No presence of flavonoids was detected in the control bread, while the application of only 5% apple pomace resulted in a significant content of 8.04 mg rutin/100 g d.m. (Table 3). In the case of the GFB15AP sample, the content of flavonoids was 21.56 mg rutin/100 g d.m. Such high amounts of flavonoids being determined in gluten-free bread is a result of their abundance in apple pomace (Table 1 and Table 3).

Apart from the application of the spectrophotometric method, the individual phenolic compounds were identified using the UPLC-PDA-MS/MS method, and the results are shown in Table 4. The high contents of phenolic acids in gluten-free bread with apple pomace were accompanied by the occurrence of only a few such compounds in the applied additive, namely chlorogenic, cryptochlorogenic and p-coumaroylquinic acids (Table 2 and Table 4). The presence of other phenolic acids in bread could be due to the interaction of many different factors. Most probably, the profile of the phenolic acids was modified in the subsequent stages of gluten-free production. In previous studies, an increase in phenolic acids was observed during yeast fermentation [43] and dough mixing [45]. Moreover, thermal disruption of quercetin derivatives, especially quercetin-rutinoside, could generate phenolic acids [46]. Taking into account the high quantity of quercetin in dried apple pomace, this process could lead to an increase in phenolic acids in gluten-free bread. This was indirectly confirmed by the increase in most of the analyzed phenolic acids (chlorogenic acid, cryptochlorogenic acid and p-coumaroylquinic acid, caffeoyl-dihydroxyphenyl-lactaoyl-tartaric acid, 1-*O*-p-coumaroyloglycerol) accompanying an increase in the levels of added apple pomace. Only one phenolic acid was detected in smaller quantities in comparison with control (p-coumaroyl spermidins), and this was due to a thermal decarboxylation of these compounds, e.g., to 4-vinyl guaiacol [47] (Table 4). Among the analyzed samples of gluten-free bread with added apple pomace, the greatest increase in comparison to control could be found for chlorogenic acid (7 times). In the case of p-coumaroylquinic acid and caffeoyl-dihydroxyphenyl-lactaoyl-tartaric acid, 3-fold increases in their level were observed with the introduction of apple pomace into the gluten-free bread formulation.

According to Rupasinghe et al. [46], the thermal resistance of flavonols, including the above-mentioned derivatives of quercetin, is relatively higher than that of phenolic acids and anthocyanins. However, the applied baking temperature (230 °C) could cause their partial decomposition, as evidenced by the appearance of the above-mentioned phenolic acids in the gluten-free breads containing apple pomace (Table 4).

On the other hand, the content of flavan-3-ols, including catechins, as well as procyanidins in gluten-free breads containing apple pomace was relatively low, and in the case of epicatechins, the levels were below the detection limits in the above-mentioned products (Table 4). This is most probably connected with the significant decomposition of these compounds, especially catechins, resulting from a combination of several processes, including oxidation, isomerization, and epimerization occurring during baking [48] and at other stages of bread production [46]. Additionally, losses of these phenolic compounds may be caused by the formation of complexes with polysaccharides [48].

In the case of dihydrochalcons, i.e., phloretin and phloridzin, previously detected in apple pomace, a significant content of these compounds (especially the latter compound) was also found in the breads containing this addition (Table 2 and Table 4). Their amount in the gluten-free breads corresponded to the amount of the above-mentioned pomace added. The amount of phloridzin in gluten-free breads containing apple pomace increased by 21–75 times relative to the control. In the case of phloretin, the addition of apple pomace to gluten-free breads caused a 12.5-fold increase compared to the control (Table 4).

Despite the above-mentioned losses of polyphenols during baking, it should be emphasized that the addition of apple pomace (in a content range from 5 to 15%) guarantees an increase in polyphenol content in these gluten-free breads (with up to a 7-fold increase in chlorogenic acid, a 21.5–39-fold increase in quercetin derivatives, a doubling of procyanidin B2, a 4.5–12.5-fold increase in phloretin-2-*O*-xylosyl-glucoside, and a 21–75-fold increase in phloretin-2-*O*-glucoside), which translates into a high antioxidant potential of the final product (Table 3 and Table 4). Even the smallest share of apple pomace (5%) in GFB caused a 66-fold increase in antioxidant activity compared to control, and a 15% share of apple pomace resulted in a 107-fold increase in this activity (Table 3). In the study of Korus et al. [44], the increase in antioxidant activity ranged from 12 to 68% in GFB with defatted blackcurrant seeds, and from 165 to 370% in the case of the use of defatted strawberry seeds, in comparison to control. Similarly, the amount of polyphenols increased in the breads analyzed by Korus et al. that contained a share of fruit seeds [44] in comparison to the control. In the research of Zlatanović et al. [20] on cookies fortified with up to 75% apple pomace flour produced by industrial-scale dehydration, an increase in the amount of polyphenols (8–126%) and flavonoids (3–8 times) was seen in comparison to the control. Additionally, the antioxidant activity of these cookies was 3 to 5.5 times higher than that of the control cookies [20]. In the study of Mir et al., [18] regarding the influence of apple pomace on the quality of gluten-free rice crackers, an increase in polyphenol content in these crackers was also noted with increasing levels of addition (from 3% to 9%). This increase ranged from 14% to 34%, while the increase in flavonoids in the analyzed product ranged from 9% to 28%, compared to the control sample. According to Mir et al., [18], the above-mentioned changes were proportional to the addition level applied. Similarly, in this work, the increase in TPC and flavonoids was parallel to the increase in the proportion of apple pomace in GFB (Table 3). Šarić et al., [19], while studying gluten-free cookies with different proportions of blueberry and raspberry pomace, also observed a significant content of bioactive compounds following the introduction of pomace. Gluten-free cookies with a mixture of the above-mentioned pomace types in different proportions (total additive content 30%) were characterized by a 725-fold to 2500-fold higher content of total polyphenols compared to the control. Moreover, Šarić et al., [19] proved that cookies prepared with only blueberry pomace contained 6-fold more TPC than cookies with raspberry pomace. This also resulted antioxidant activity in these products many times greater than the control. The authors, similarly to Mir et al., [18], explained this huge increase in the bioactive content of cookies as being a result of the use of colored fruit pomace in the production of these products. This study also showed that using apple pomace in the production of GFB guaranteed a significant amount of polyphenols and high antioxidant activity of these breads (Table 3 and Table 4).

We suggest that, due to the high antioxidant potential of apple pomace when introduced into gluten-free bread, a high antioxidant potential of the finished product is guaranteed, which may contribute to the reduction of oxidative stress, which would affect inflammation and may protect against DNA damage, which in turn may prevent future chronic non-communicable diseases and even cancer in people with celiac disease.

It should be noted that oxidative stress is significantly higher in patients shortly after diagnosis, who have not yet started a gluten-free diet [9]. The findings suggest that it is gliadin that disrupts the pro-oxidant–antioxidant balance in the small intestinal mucosa of affected individuals through overproduction of ROS [49]. Additionally, in vitro studies have shown redox imbalance and increased free radical levels after exposure of cells to gliadin [50]. Stojiljković et al., [7] showed that oxidative stress is strongly associated with CD, and that antioxidant capacity in celiac patients is impaired by glutathione (GSH) depletion and reduced activity of glutathione peroxidase and glutathione reductase (GPx and GR), as well the activity of other enzymes, including CuZn SOD and Mn SOD. It has also been shown that the greater the oxidative stress in people with celiac disease, the more advanced the mucosal damage is. Therefore, a diet rich in natural antioxidants may be beneficial for complete mucosal healing in celiac patients [7].

We suggest that the product obtained in this work, i.e., GFB with apple pomace, seems to be an example of a product that could increase the supply of antioxidants in a gluten-free diet, in which bread, based only on starch and hydrocolloids, is a key element. Therefore, the obtained GFB with apple pomace could be recommended especially at the beginning of a gluten-free diet, because at this point, it seems reasonable to introduce products rich in antioxidants.

As has already been mentioned, among all of the analyzed samples of gluten-free breads containing apple pomace, the GFB15AP sample was characterized as having the highest antioxidant activity. The high antioxidative potential of the above-mentioned bread is reflected by its having the highest content of total polyphenols, the largest quantity of flavonoids, determined by spectrophotometric methods, as well as the amounts of individual phenolic acids, dihydrochalcones and some flavonols (especially quercetin derivatives) determined using the UPLC-PDA-MS/MS method (Table 3 and Table 4). In this study, a correlation between TPC and ABTS was observed (r = 0.84). Nevertheless, it should be remembered that individual phenolic compounds have a specific antioxidant activity. Among the above-mentioned compounds, high antioxidant activity has been demonstrated for quercetin derivatives and chlorogenic acid [51]. This is reflected by the correlation coefficients between this acid and ABTS (r = 0.95) and the sum of flavonols (as derivatives of quercetin) and ABTS (r = 0.94). High correlation coefficients between the above-mentioned compounds and antioxidant activity confirm the observations of earlier authors, who observed a strong correlation between phenolic acids, flavonols and antioxidant activity [51,52].

Gluten-free breads enriched with antioxidants from the polyphenol group derived from the apple pomace guarantee high health-promoting potential. It is known that polyphenols have anticancer, anti-inflammatory, antibacterial, and antiviral properties [12,13]. The polyphenols that were detected in this type of bread, i.e., chlorogenic acid, quercitin derivatives, catechins, procyanidin and dihydrochalcone (phloridzin), are especially valuable [35,36,37,38,53,54,55],

The high antioxidant potential of breads with apple pomace that is confirmed in this work should be verified in in the context of a diet for celiac patients in further research.

### 3.3. Organoleptic Analysis of Gluten-Free Bread Enriched with Apple Pomace

**Figure 1:**
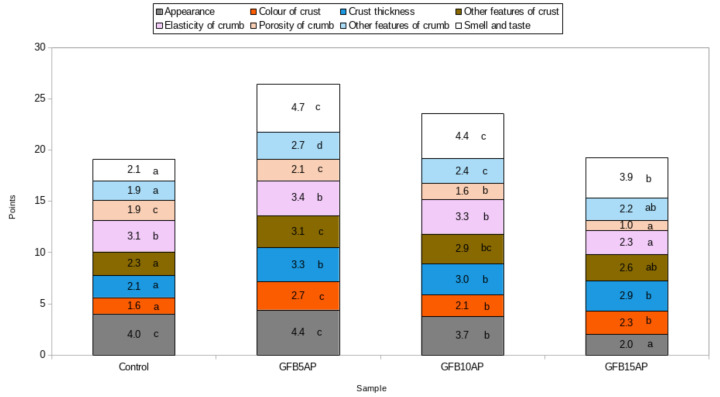
Organoleptic evaluation of gluten-free breads with apple pomace (values signed the same letters a–d in particular colors are not significant at 0.05 level of confidence).

The appearances of the control bread and the bread with the 5% share of apple pomace were judged to be the best. The other two breads, with higher shares of apple pomace, obtained significantly lower ratings (Figure 1). The crust color of breads is an important parameter affecting consumer acceptance [56]. The crust color of the control bread was rated significantly lower in comparison to the breads containing apple pomace. The evaluations of this parameter in the breads containing 10% and 15% apple pomace did not differ from each other, while the loaf with the lowest apple pomace content (5%) received the highest rating, which was significantly different from the other breads. This may have been due to the darker crust color, resulting from the addition of apple pomace, while at higher contents of this ingredient, the crust was rated as being too dark. A significant crust darkening was observed by Torbica et al., in wholegrain wheat bread with 10% coextrudate of apple pomace and corn grits [57]. Additionally, Rocha Parra et al., [58] found a decrease in the L* parameter value, indicating darkening of both the crust and the crumbs of gluten-free breads based on rice flour and cassava starch with increasing amounts of apple pomace.

The addition of 5 and 10% apple pomace did not significantly affect the crumb elasticity of the gluten-free breads compared to the control bread. However, the highest addition of apple pomace, 15%, significantly decreased the elasticity of the bread, as judged by the panelists (Figure 1). Bchir et al., [56] found no significant differences in instrumentally tested crumb elasticity between the control wheat bread and breads with apple fiber from cooked by-products. With respect to crumb porosity, only 5% apple pomace did not significantly affect this characteristic in comparison with control bread. On the other hand, crumb porosity was rated significantly lower for higher proportions of apple pomace (Figure 1).

The most important features in organoleptic evaluation are the taste and smell of the product, which are the factors most responsible for consumer acceptance. In the case of the tested gluten-free breads, the apple pomace significantly increased the evaluation of the taste and smell in comparison with the control bread, irrespective of the amount of this ingredient added. The breads with 5 and 10% addition of apple pomace did not differ from each other with respect to the evaluation of this parameter, while the highest addition level (15%) showed a decrease in this parameter, although it was, however, still significantly higher than the case of the control bread. These data corroborate the study of Jannati et al. [59], who studied the effect of apple pomace addition of 1–7% on wheat flour Sangak bread. They observed a significant increase in the organoleptic evaluation scores for the smell for all breads containing apple pomace. In contrast, the score for taste was not significantly different from the control bread, except for the bread with 3% apple pomace, which was scored the best.

The organoleptic evaluation showed that the share of apple pomace positively influenced the results obtained in the evaluation of breads (Figure 1). Among the samples containing apple pomace, the bread with the lowest, 5%, share of pomace received the best evaluation; it received the highest scores for external appearance, the color of the crust, and other crumb characteristics, as well as other features of crust and smell and taste.

## 4. Conclusions

In summary, the bread with 15% apple pomace was the best in terms of possessing the highest content of marked phenolic compounds (total phenolic content, total flavonoids, phenolic acids, dihydrochalcons, and flavonols, especially quercetin derivatives) and antioxidant potential among the gluten-free breads with apple pomace analyzed in this study. Unfortunately, this bread received the lowest organoleptic scores. The best results of organoleptic analysis were determined for the bread with a 5% share of apple pomace. Bread with this level of apple pomace still has 2.5 times more polyphenols, 8 times more flavonoids, 4 times more chlorogenic acid and 21 times more phloridzin than the control, resulting in 6.5 times higher antioxidant potential. Therefore, it can be recommended as an innovative gluten-free product for people with gluten intolerance that should be produced on an industrial scale. We then suggest that this type of bread may help in reducing oxidative stress affecting inflammation, and may protect against DNA damage. Therefore, it may reduce the occurrence of non-communicable diseases, including even cancer, in patients with celiac disease, which should be verified through further studies.

## Figures and Tables

**Table 1:** Antioxidant compounds and antioxidant activity in apple pomace.

By-Product	Total Phenolic Content (mg Gallic Acid/100 g d.m.)	Total Flavonoids Content (mg Rutin/100 g d.m.)	Trolox Equivalent Antioxidant Capacity (mg Tx/g d.m.)
Apple pomace (AP)	89.4	94.3	9.30

**Table 2:** Quality and quantity of phenolic compounds in the apple pomace.

Compounds		Content in Apple Pomace (mg/100 g d.m.)
Flavonols	luteolin 6-C-hexoside *O*-hexoside	n.d.
	luteolin *O*-hexoside C-hexoside	n.d.
	quercetin-*O*-rutinoside	2.82 ± 0.02
	quercetin-3-*O*-galactoside	22.55 ± 0.34
	quercetin-3-*O*-glucoside	5.88 ± 0.10
	quercetin-3-*O*-arabinoside	8.77 ± 0.27
	quercetin-3-*O*-xyloside	13.91 ± 0.03
	quercetin-3-*O*-rhamnoside	19.21 ± 0.00
	isorhamnetin-3-*O*-galactoside	0.74 ± 0.00
	isorhamnetin-3-*O*-glucoside	0.57 ± 0.00
Phenolic acids	chlorogenic acid	20.55 ± 0.12
	cryptochlorogenic acid	1.03 ± 0.00
	caffeoylquinic acid	n.d.
	p-coumaroylquinic acid	0.16 ± 0.03
	caffeoyl-dihydroxyphenyl-lactaoyl-tartaric acid	n.d.
	2-*O*-p-coumaroylglicerol	n.d.
	1-*O*-p-coumaroylglicerol	n.d.
	p-coumaroylspermidin	n.d.
	di-p-coumaroylspermidin	n.d.
	ferullyquinic acid	n.d.
Flavon-3-ols	(+) catechin	1.44 ± 0.02
	procyanidin B2	2.61 ± 0.00
	(−) epicatechin	0.76 ± 0.00
Dihydrochalcones	phloretin-2-*O*-xylosyl-glucoside	1.48 ± 0.14
	phloretin 2-*O*-glucoside (phloridzin)	15.52 ± 0.00

n.d.—not detected; ±—standard deviation.

**Table 3:** Antioxidant compounds and antioxidant activity in gluten-free breads with apple pomace.

Sample	Total Phenolic Content (mg Gallic Acid/100 g d.m.)	Change to Control	Total Flavonoids Content (mg Rutin/100 g d.m.)	TEAC (mg Tx/g d.m.)	Change to Control
Control	1.02 ± 0.00 a *	-	n.d.	0.03 ± 0.00 a *	-
GFB5AP	3.58 ± 0.00 b	250%	8.04 ± 0.10 b	1.97 ± 0.19 b	6467%
GFB10AP	7.15 ± 1.57 c	600%	15.87 ± 0.27 c	2.26 ± 0.05 c	7433%
GFB15AP	21.96 ± 2.00 d	2050%	21.56 ± 0.31 d	3.21 ± 0.10 d	10600%

* Presented data are mean values ± standard deviation (values signed the same letters (a–d) in particular columns are not significant at 0.05 level of confidence). TEAC—Trolox Equivalent Antioxidant Capacity; n.d.—not detected.

**Table 4:** Quality and quantity of phenolic compounds (mg/100 g d.m.) in gluten-free breads with apple pomace.

Compounds		Control	GFB5AP	GFB10AP	GFB15AP
Flavonols	luteolin 6-C-hexoside *O*-hexoside	0.84 ± 0.20 a *	0.99 ± 0.07 a	0.97 ± 0.00 a	1.07 ± 0.03 b
	luteolin *O*-hexoside C-hexoside	0.96 ± 0.00 c	1.00 ± 0.08 c	0.88 ± 0.00 b	0.82 ± 0.02 a
	quercetin-*O*-rutinoside	n.d.	0.22 ± 0.03 a	0.44 ± 0.01 b	0.52 ± 0.05 c
	quercetin-3-*O*-galactoside	0.11 ± 0.02 a	1.21 ± 0.17 b	2.60 ± 0.09 c	4.37 ± 0.00 d
	quercetin-3-*O*-glucoside	0.01 ± 0.00 a	0.25 ± 0.00 b	0.63 ± 0.00 c	1.02 ± 0.07 d
	quercetin-3-*O*-arabinoside	0.08 ± 0.00 a	0.47 ± 0.03 b	0.93 ± 0.02 c	1.72 ± 0.05 d
	quercetin-3-*O*-xyloside	0.10 ± 0.01 a	0.83 ± 0.03 b	1.49 ± 0.00 c	2.89 ± 0.07 d
	quercetin-3-*O*-rhamnoside	0.13 ± 0.02 a	1.06 ± 0.07 b	2.00 ± 0.00 c	3.71 ± 0.00 d
	isorhamnetin-3-*O*-galactoside	n.d.	n.d.	0.10 ± 0.03 a	0.21 ± 0.00 b
	isorhamnetin-3-*O*-glucoside	n.d.	n.d.	0.14 ± 0.01 a	0.15 ± 0.00 a
Phenolic acids	chlorogenic acid	0.35 ± 0.00 a	1.33 ± 0.09 b	2.36 ± 0.00 c	3.74 ± 0.12 d
	cryptochlorogenic acid	n.d.	0.06 ± 0.00 a	0.12 ± 0.00 b	0.19 ± 0.04 c
	caffeoylquinic acid	0.42 ± 0.00 a	0.49 ± 0.03 b	0.37 ± 0.06 a	0.57 ± 0.00 c
	p-coumaroylquinic acid	0.07 ± 0.00 a	0.13 ± 0.00 b	0.21 ± 0.02 c	0.32 ± 0.01 d
	caffeoyl-dihydroxyphenyl-lactaoyl-tartaric acid	0.15 ± 0.00 a	0.28 ± 0.00 b	0.41 ± 0.00 c	0.61 ± 0.00 d
	2-*O*-p-coumaroylglicerol	0.28 ± 0.00 ab	0.26 ± 0.02 ab	0.25 ± 0.00 ab	0.23 ± 0.01 a
	1-*O*-p-coumaroylglicerol	1.39 ± 0.00 a	1.74 ± 0.06 c	1.58 ± 0.00 b	1.54 ± 0.01 b
	p-coumaroylspermidin	0.27 ± 0.05 d	0.16 ± 0.01 c	0.11 ± 0.00 b	0.05 ± 0.00 b
	di-p-coumaroylspermidin	0.98 ± 0.03 a	1.21 ± 0.12 b	1.10 ± 0.01 b	1.12 ± 0.03 b
	ferullyquinic acid	0.16 ± 0.00 a	0.31 ± 0.01 b	0.35 ± 0.01 b	0.34 ± 0.02 b
Flavon-3-ols	(+) catechin	0.09 ± 0.00 a	n.d.	0.15 ± 0.01 b	n.d.
	procyanidin B2	0.20 ± 0.00 a	0.32 ± 0.00 c	0.28 ± 0.00 b	0.46 ± 0.00 d
	(−) epicatechin	n.d.	n.d.	n.d.	n.d.
Dihydrochalcones	phloretin-2-*O*-xylosyl-glucoside	0.02 ± 0.00 a	0.09 ± 0.00 b	0.16 ± 0.00 c	0.25 ± 0.01 d
	phloretin 2-*O*-glucoside (phloridzin)	0.04 ± 0.00 a	0.84 ± 0.00 b	1.78 ± 0.03 c	2.99 ± 0.02 d

* Presented data are mean values ± standard deviation (values signed the same letters a–d in particular lines are not significant at 0.05 level of confidence). n.d.—not detected.

## Data Availability

All data concerning the presented studies are included in the paper.
